# Dose-response of resistance training for neck-and shoulder pain relief: a workplace intervention study

**DOI:** 10.1186/s13102-020-0158-0

**Published:** 2020-04-01

**Authors:** Atle Hole Saeterbakken, Paula Makrygiannis, Nicolay Stien, Tom Erik Jorung Solstad, Matthew Shaw, Vidar Andersen, Helene Pedersen

**Affiliations:** Faculty of Education, Arts and Sports, Western Norway University of Applied Sciences, Sogndal, Norway

**Keywords:** Visual analog scale, Quality of life, Strength training, Office works

## Abstract

**Background:**

Musculoskeletal disorders are highly prevalent among office workers, with strong evidence suggesting that workplace-based resistance training programs can prevent several upper extremity musculoskeletal disorders. The aim of the present study was to examine the dose-response relationship between resistance training frequency and pain relief among office workers with neck- and shoulder pain.

**Methods:**

Thirty participants with mild to moderate neck- and shoulder pain attended a 16-week intervention starting with an eight-week control period followed by an eight-week training period. After the control period, the participants were randomized into either a 10 min (TG10) or 2 × 10 min (TG2) workplace-based, high-intensity neck- and shoulder specific resistance training program that was executed 5 days per week and consisting of four exercises. The participants were tested pre and post each period for mean and worst pain using the 0-100 mm visual analog scale (VAS), 0-100 mm health-related quality of life and isometric strength of the neck-and shoulder region. The analysis of variance (ANOVA) and Friedman with Bonferroni post hoc corrections were used to assess differences in between and within groups for the three testing times pre, mid and post intervention.

**Results:**

No differences were observed between the groups in any of the variables in the control period (*p* = 0.27–0.97) or training period (*p* = 0.37–0.68). When merging the two groups, the mean and worst pain was reduced by 25 and 43% (*p* = 0.05 and < 0.01, ES = 0.41 and 0.55) in the training period in addition to 10.6% increase in health-related quality of life (*p* = 0.01, ES = 0.52). No difference in strength was observed (*p* = 0.29–0.85).

**Conclusion:**

Daily bouts of specific high-intensity resistance training of the shoulder and neck region at the workplace reduced neck- and shoulder pain and improved quality of life of office workers. However, 10 min bouts were equally effective as 2 × 10 min bouts per day. The authors recommend office workers to perform daily neck- and shoulder resistance training to possibly prevent and/or decrease pain in the neck- and shoulder area.

**Trial registration:**

ISRCTN69968888, retrospectively registered (24/09/2019).

## Background

Neck- and shoulder pain is the second most common musculoskeletal disorder, with more than half of all adults reporting having experienced neck- and shoulder pain the last six months [1, 2]. Musculoskeletal disorder is more prevalent among office workers performing low intensity, but continuous, isometric contraction in the neck- and shoulder region (e.g. computer work, hairdresser or dentist) [3–5]. A recent systematic review concluded that there was strong evidence that workplace-based resistance training programs can prevent several upper extremity musculoskeletal disorders [6]. However, high quality and long-term intervention studies are needed to provide effective training strategies and recommendations to treat and prevent neck- and shoulder pain [7].

In the last decade, several treatments have been examined. General aerobic endurance activities (i.e. cycling or walking) and more specific activities, targeting the neck- and shoulder muscles (i.e. Nordic walking and shoulder endurance exercises) [8–11], have demonstrated a reduction in pain [11–15]. Increased blood flow and temperature in the painful areas, release of adrenal hormones and decreased muscle tension have previously been proposed as explanations for the pain reduction following aerobic activities [16], however the mechanisms are still unknown. Specific resistance training of the neck- and shoulder muscles have demonstrated promising results [4, 8, 17–19] and proven to be more effective than aerobic exercises [20]. Specific resistance training has proven effective in the reduction of muscle tension [21, 22], headache [23], pain [8, 20, 24], pain perception [25] and improvements in strength [5, 20]. In recent years, as little as 2 minutes of high-intensity resistance training per day has demonstrated increased strength and torque, improved muscle relaxation and reduced pain among office workers with neck- and shoulder pain [4, 22].

In rehabilitation, scientists and therapists attempt to quantify a relationship between dose (training) and response (pain relief). The dose-response relationship is vital for prescribing optimal and efficient training for pain relief whilst avoiding over-or-under prescription. Nonetheless, the dose-response relationship between pain relief and resistance training is not conclusive [18, 25–27]. Nikander et al. [26] demonstrated that upper extremity training of more than 8.75 metabolic equivalent task (MET) hours per week reduced pain among patients with neck pain. Furthermore, Andersen et al. [25] examined pain perception following resistance training for the neck-and shoulder muscles using different training volume per day, but equal training frequency per day. The training groups performed either 2 or 12 min, 5 days per week, and increased their respective pain thresholds, but no differences were observed between them [25]. The same research group also compared similar overall training (approximately 60 min per week) among office workers with neck –and shoulder pain [18]. However, the training was performed with different training frequency during a week (i.e. as one session of 60 min, three session of 20 min or seven sessions of 9 min). Similar pain relief was observed between the groups [18]. The pain relief using similar training volume per session, but different training sessions per week, was examined in females with severe neck pain [27]. One to two sessions per week (20 min per session) over a 20-week training period demonstrated superior effects compared to 0–1 session per week and a passive control group, but similar effects as 2–3 sessions per week [27].

Studies trying to find the best dose of training to optimize the pain relief in neck- and shoulder patients, has examined different weekly training frequencies, different volumes or similar volumes but divided into long and short sessions [18, 25, 27]. However, it is not clear whether different training volumes, with different frequencies of resistance training per day, modifies the dose-response relationship concerning increased pain relief. Therefore, the aim of the present study was to examine the dose-response relationship between resistance training frequency and pain relief among office workers with neck- and shoulder pain. The participants attended either a 10 min or 2 × 10 min workplace-based specific resistance training program, 5 days per week. It was hypothesized that both training programs would reduce pain and improve strength with, greater pain relief in the 2 × 10 min group.

## Methods

### Study design

**Fig. 1:**
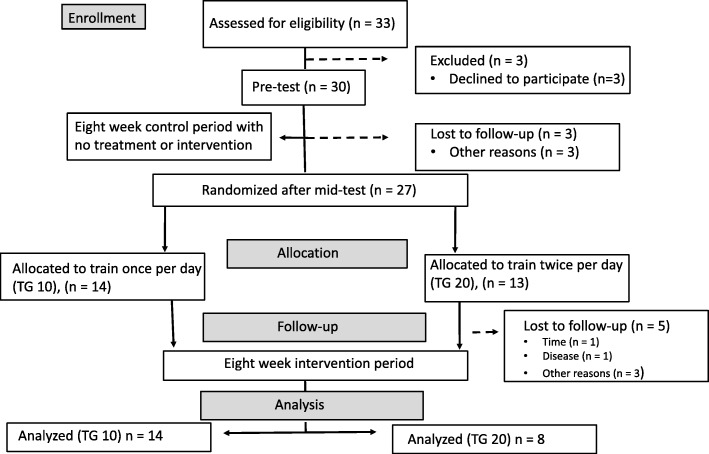
The flowchart of the study

The study was a training intervention starting with an eight-week control period. The participants were then randomized into either a training group performing 10 min (TG10), or a group performing 10 min twice per day (TG20), for 8 weeks (Fig. 1). The training was conducted five times per week in the participants’ workplace. In the control period, the participants were instructed to continue their normal activities. The participants were tested before the control period (pre-test), between the control- and training period (mid-test) and after the training period (post-test). The testing included the 0-100 mm visual analog scale (VAS) for pain (primary outcome), isometric strength in shrugs and seated row (secondary outcome) and health-related quality of life (secondary outcome).

### Subjects

The study was planned to detect a moderate to large effect size (> 0.3) for pain relief (primary outcome) defined as a clinical effect [28]. With a statistical level set to 0.05, the statistical power to 80%, and using the pain relief from comparable studies [8, 20], 14 participants were required to significant difference. An e-mail with information about the study was sent to public workplaces with typical office workspaces in the region Sogn og Fjordane, Norway. To be included, participants should have mild to moderate pain (10 – 60 mm VAS) [8, 18, 24] in the neck and/or shoulder region lasting at least 3 months and having computer work or low-intensity isometric contraction during work (i.e. dentist, hairdresser). Thirty-three respondents (26 women and 7 men) volunteered to participate in the study, but only 30 attended the pre-testing (23 women and 7 men). Among these, three were hairdressers, six were dentists and 21 were office workers with computer work as their main task). People with considerable pain (> 60 mm VAS) was excluded as a resistance training can cause increased acute pain following the session [13]. In addition, participants receiving treatment the last 6 months by health care professionals were also excluded. After the mid-test, participants were randomized to train either 10 min per day (TG10) or 10 min twice per day (TG20). Three participants withdrew during the control period and five withdrew during the intervention period for reasons not related to the study. The details of the groups are presented in Table 1.

**Table 1:** An overview of the participants demographics at baseline

	Total	TG 10	TG 20
Total (n) ♀ /♂	27 (20 / 7)	14 (11 / 3)	13 (9 / 4)
Age (years)	48.7 ± 11.8	50.1 ± 12.9	46.2 ± 9.7
Height (cm)	170.8 ± 6.5	170.1 ± 6.9	172.1 ± 5.8
Weight (kg)	72.5 ± 9.2	74.0 ± 10.1	70.1 ± 7.3
BMI* (kg/m^2^)	24.9 ± 3.6	25.6 ± 3.9	23.7 ± 2.7

*♀* women, ♂ men, *BMI* Body mass index, *TG 10* training 10 min per day, *TG 20* training 10 min twice per day

### Ethical statement

All participants were informed orally and in writing before giving their written informed consent to participate. All participants could withdraw from the study at any time without giving a reason. The study was approved by the local regional ethics committee (2016/1280 Sør-øst B) and conformed to the latest version of the Declaration of Helsinki. The study was retrospectively registered in the ISRCTN registry (69968888).

### Procedures

The training groups trained one (TG10) or two (TG20) sessions, 5 days per week, across 8 weeks. Each session lasted 10 min. For the first week (5 sessions), a personal trainer was present. After the first week, the participants conducted the exercises independently. The instructor visited the workplaces every week to conduct and monitor one training session with the participants. A questionnaire including pain (worst and general), health-related quality of life and training attendance was also conducted each week. The TG20 group were encouraged to train at the beginning and end of the working day whilst the TG10 group trained at the time that best suited them. The TG10 and TG20 groups reported to perform 89 and 87% of the training sessions respectively.

### Training

**Fig. 2:**
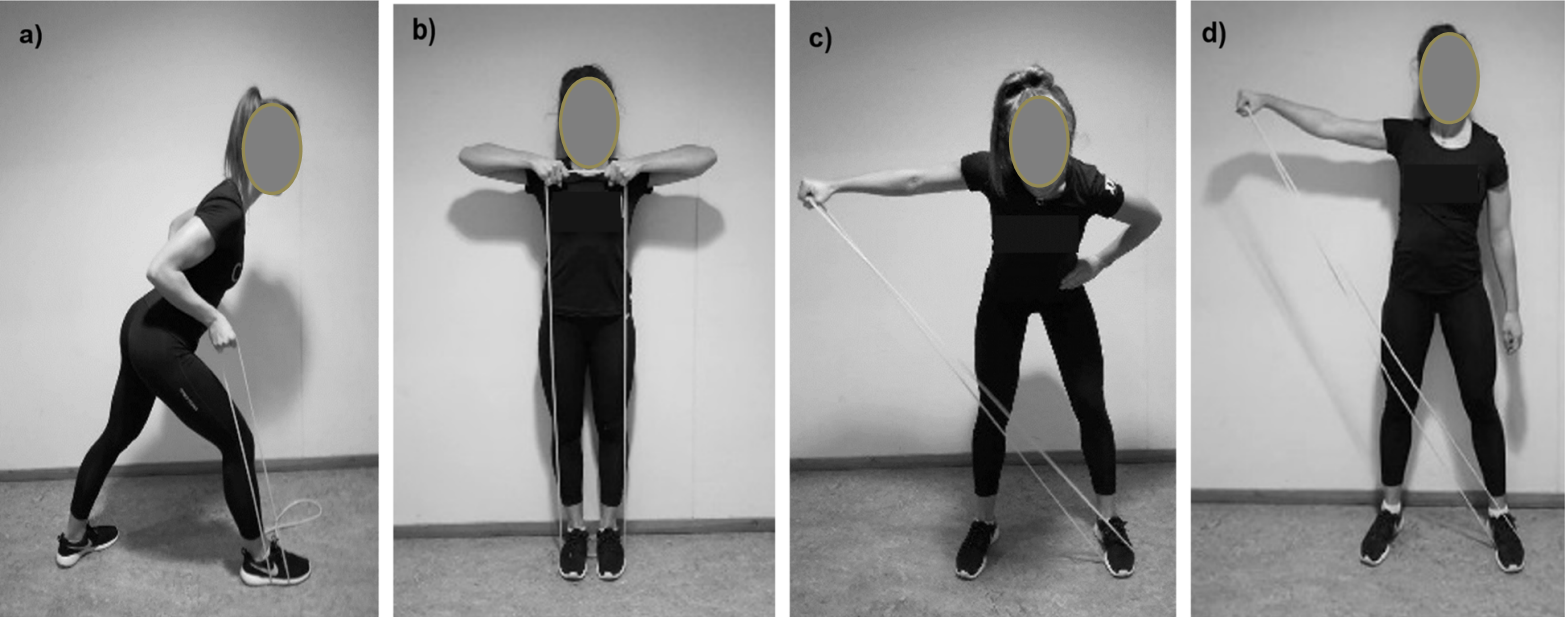
**a**-**d**. The exercises used in the training **a**) one-arm row exercise, **b**) upright row exercise, **c**) one-arm revers flies exercise and **d**) one-arm lateral raise exercise

The exercises were conducted without any specific warm-up procedures. Each session consisted of four specific neck-and shoulder exercises using elastic tubes [8, 29, 30]: one-arm row (Fig. 2a), upright row (Fig. 2b), one-arm reverse flies (Fig. 2c) and one-arm lateral raise (Fig. 2d). Each exercise was conducted with two sets. The intensity was 12–15 repetition maximum (RM) in the first 4 weeks and 8-10RM in week 5–8 [9, 18].. During the first week of the training intervention, an experienced instructor was present to instruct the participants to add the correct resistance from the tubes. If the participants performed more or less repetitions than prescribed, they were instructed to adjust the intensity. The progression of the resistance was typically implemented in the following order of 1) shortening the tube in the starting position of the exercise, 2) use of thicker tubes 3) use of thicker tubes and shortening the length and 4) use of two tubes [8, 31] (see Fig. 2a-d). Two types of elastic tubes (ROPES AS, Aasgaardstrand, Norway) were used to provide resistance. The resistance from the tubes were 40 N and 54 N stretched 150% of their resting length.

All exercises were performed during standing, with a controlled speed and no rest between repetitions. In the one-arm row exercise (Fig. 2a), the participants had 45° flexion in the hip. In the starting position, the participants stood on their elastic tube with the elbow fully extended (starting position). When the hand touched the chest, the elbow was extended to the starting position. The participants were instructed to press the shoulder blade medial with a relaxed and depressed shoulder. In the upright row exercise (Fig. 2b), the participants held on to the elastic tube with both hands. The arms were abducted with flexed elbows. The participants returned to the starting position (fully extended elbows with the arms along the side of the body) after the arms had been elevated to the upper sternum height. In the one-arm reverse flies (Fig. 2c), the participants had 45° flexion in the hip with the contralateral foot on the elastic tube. The arm was abducted from a vertical position to a horizontal position. The elbow had 170° flexion (180° = fully extended). In the one-arm lateral raise exercise (Fig. 2d), the participants stood on the contralateral foot with extended hip (standing straight up). The arm was abducted from a vertical position (starting position) to a horizontal position with 170° flexion (180° = fully extended) elbow.

### Measurements

#### Pain and health-related quality of life

The 100 mm visual analog scale (VAS) was used to examine general pain and worst pain, twice per week (Tuesday and Friday). Zero indicated “no pain at all” whereas 100 indicated “worst possible pain” [9, 17, 24]. In addition, a questionnaire (EQ-5D-5 L) was used to examine the health-related quality of life [32]. The participants were asked to mark health-related quality of life today on 0–100 scale. Zero was defined as “the worst possible health” and 100 as “the best possible health”.

#### Isometric strength

**Fig. 3:**
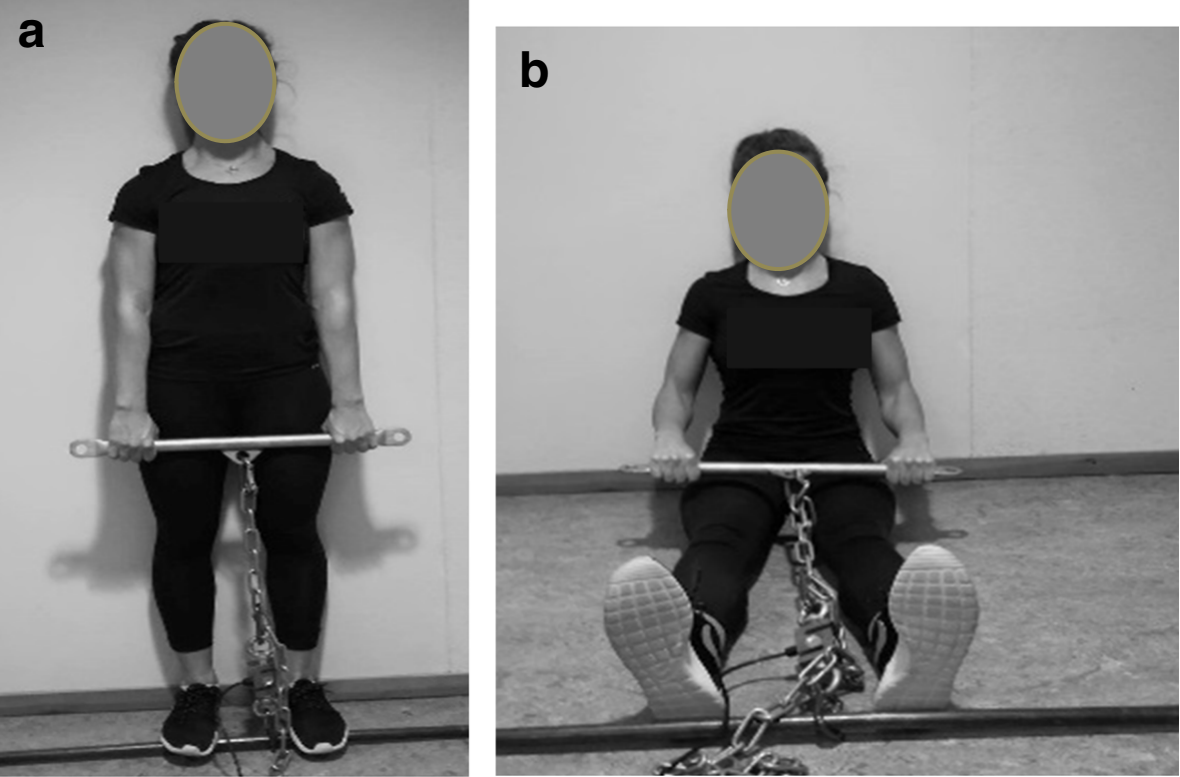
**a**-**b**. The isometric testing procedures performing the shrugs (**a**) and seated row (**b**)

Two maximal voluntary isometric contraction (MVIC) tests were used to examine strength in the neck- and shoulder region. The exercises were shrugs and seated row [24, 29]. In the shrugs exercise, the participants stood upright along a wall to avoid hip extension, with a chain connected to the force cell (Ergotest Technology AS, Langesund, Norway) and the barbell (Fig. 3a). The length of the chain was adjusted for each participant so that the participants’ shoulders were in a natural and relaxed position. The participants were instructed to elevate their shoulders without extending the hip, legs or arms. In the seated row exercise, the participants sat against a wall with a 90° angle in the elbow and hip (Fig. 3b). A chain connected the force cell and the barbell. The length of the chain was individually adjusted. The participants were instructed to press the shoulder blades together and elbows backward without elevating the shoulders or flexion the wrists.

The force output was measured using a force cell (KTOYO, Model 33A CAP 500, S/N 10038) from Ergotest Technology AS (Langesund, Norway). The force cell was attached to the synchronization unit Muscelab 4020e (Ergotest Technology AS, Langesund, Norway). Three attempts were performed for each exercise, separated by approximately 60 s [24]. The participants were instructed to gradually increase the force and maintain the maximal voluntary contraction for 5 seconds and the highest mean force output during a three-second window was used in further analyses [31].The best of three attempts was used in further analyses [8]. The intra class correlation coefficient between three attempts in the pre-test was 0.984 and 0.860 for shrugs and seated row.

### Statistical analyses

A one-way analysis of variance (ANOVA) and dependent t-test was used to assess differences in strength (shrugs and row exercises) between and within groups for the three testing times pre, mid and post intervention. For the non-parametric tests (pain and health-related quality of lift), Friedman and Wilcoxon signed rank tests were used. In the ANOVA and Friedman, Bonferroni post hoc corrections were used to assess differences in between and within groups for the three testing times pre, mid and post intervention. Where significant differences were observed for the parametric tests, Cohens d effect size (ES) was calculated. For the non-parametric tests, the z-score divided by the square root of the total sample size was used (r statistic). An ES of 0.2 was considered small, 0.5 medium and 0.8 large [33] whereas *r*-values of < 0.3 was consider small, 0.3–0.5 medium and > 0.5 large. All parametric data are presented as mean ± standard deviation. For the non-parametric data, the median and the 25–75 percentile interquartile range are presented. All statistical analyses were conducted with SPSS version 25.0 (SPSS, Inc., Chicago, IL, USA). Statistical significance was accepted at *p* ≤ 0.05.

## Results

There were no differences between the TG10 and TG20 in pain (general and worst), health-related quality of life or isometric strength (shrugs or rowing) in the control period (between pre and mid-test) (*p* = 0.27–0.97) or in the training period (between mid and post-test) (*p* = 0.37–0.68).

Since there was no difference between the training groups the groups were merged to examine whether a workplace intervention could be effective to improve neck- and shoulder pain health-related quality of life and strength. For general pain, no change was observed in the control period (*p* = 0.43), but a 25% reduction in pain in the training period (*p* = 0.05, ES = 0.41; see Table 2).

**Table 2:** The pain (general and worst) and health-related quality of life (HRQL). All values are presented as the median and the 25th – 75th percentile

	Pre	Mid	Post
General pain (mm)	20.0	20.0	15.0*
25th – 75th percentile	(15.0–30.0)	(10.0–35.0)	(5.0–21.3)
Worst pain (mm)	40.0	35.0	20.0*
25th – 75th percentile	(15.0–55.5)	(20.0–40.0)	(8.6–30.0)
HRQL (mm)	80.0	75.0	88.5*
25th – 75th percentile	(68.0–85.0)	(70.0–90.0)	(80.0–95.8)

*significant difference between mid- and post-test (*p* < 0.05)

For the worst pain, no changes in the control period (*p* = 0.57). In the training period, a 43% reduction in pain was observed (*p* < 0.01, ES = 0.55; see Table 2).

For the health-related quality of life, no changes in the control period (*p* = 0.76), but a 10.6% improvement in the training period (*p* = 0.01, ES = 0.52; see Table 2).

For isometric strength measured in the exercises shrugs and seated rowing, no difference between the testing times were observed (*p* = 0.29–0.77 and *p* = 0.32–0.85). For details, see Table 3.

**Table 3:** The isometric strength between the three testing times

	Pre	Mid	Post	*p*-value
Shrugs (N)	743.9 ± 237.2	726.6 ± 203.8	750.4 ± 160.5	0.29–0.77
Seated row (N)	463.8 ± 132.9	442.2 ± 114.0	439.3 ± 84.4	0.32–0.85

## Discussion

The main findings were that no dose-response relationship between pain relief and resistance training frequency per day was observed. However, daily resistance training reduced neck- and shoulder pain in addition to increasing health-related quality of life. There were no differences in any of the variables between training 10 min or 2 × 10 min per day.

No differences were demonstrated between the two groups despite the TG20 performing twice as much training as TG10. Small sample size, recruitment of participants with only mild to moderate pain, a short training period, and subjective control of the training intensity may explain the findings. Still, previous studies using the same sample size [22], same pain intensity at baseline [18] and the same training intensity [20] have demonstrated decrease in neck – and shoulder pain in comparable studies. However, the total training volume per week for the TG10 was 50 min whereas the training volume was 100 min per week or the TG20. Previous studies have demonstrated reduction in moderate pain (40–60 mm VAS) and mild pain (10–30 mm VAS) after 60 min of specific resistance training [8, 18, 24]. It could be speculated that a training volume close to 60 min per week of high-intensity specific resistance training of the neck-and shoulder area is sufficient to reduce pain and that further training may not necessarily gain additional effects [26].

Theoretically, short intense training sessions repeated through a day, may improve the restitution, reduce muscle tension, increase temperature and blood flow in the painful muscles when compared to longer sessions. For example Andersen et al. [13] demonstrated reduced pain immediately after a training session. Improved strength in the neck-and shoulder region has proven important to prevent, but also reduce neck- and shoulder pain [13, 18]. In addition, shorter session repeated through a working day may be easier to implement and cause greater adherence than longer lasting session. This may be one way to increase the overall training volume and stress in the muscles which is essential for morphological adaptions [34].

In contrast to the present study, Andersen et al. [27] demonstrated a dose-response relationship between pain relief and training adherence whereby 1–2 sessions of 20 min yielded superior effects compared to both 0–1 session per week and no training. However, no further pain relief was elicited from performing 2–3 sessions per week. Importantly, the participants had severe pain (> 50 mm VAS) and could be the reason why similar pain relief was observed between the 1–2 sessions vs. 2–3 session per week. Shorter time to recover caused by greater weekly training session frequency may result in over-prescription among participants without resistance training experience and with painful muscles. This may explain the lack of dose-response relationship between 1 and 2 sessions vs. 2–3 sessions. Supported by the present findings, no dose-response relationship between was observed by another study conducted by Andersen et al. [25] who compared two training volumes (2 vs 12 min) which was a greater difference in training volume than the present study. Training 2 min or 12 min of high-intensity resistance training five times per week demonstrated 63 and 50% reduction in pain, respectively, without any differences between the two groups.

In addition to total training volume per week, the distribution of sessions may affect the outcomes. In the present study, short intensive 10 min sessions were conducted once (TG10) or twice (TG20) per day in the working days (i.e. five times per week). Interestingly, Andersen et al. [18] demonstrated no difference in pain relief training either 60 min once per week, 20 min three times per week or 9 min 7 times per week among 447 office workers. It may therefore be speculated that the total training volume per week is more important than the frequency of sessions per week.

As hypothesized, specific resistance training demonstrated reductions of general and worst pain when combining the training groups, with a medium effect (*r* = 0.41) in general pain and a large effect in worst pain (*r* = 0.55). Although no differences were observed between the groups, the findings are still meaningful. For example a reduction of 10 mm has been considered as clinically meaningful [28]. Importantly, the general pain was only mild and the potential to being almost pain free is not likely after the training period. However, the worst pain closer to moderate pain [35] with a greater potential of pain relief. This is most likely the reason why in the present study only the worst pain exceeded 10 mm reduction. In addition, the percentage reduction in pain was close to similar (~ 60%) for both the general and worst pain which highlights the meaningfulness of the findings. Importantly, the control period before the intervention demonstrated no change in pain (general and worst), addressing the importance of taking action when pain is experienced. The findings were supported by previous findings [4, 8, 17–19]. However, and in contrast to some of the previous studies [4, 20], no improvements in strength was observed in any of the two groups. Improved strength in the shoulder- and neck muscles has proven effective to prevent and reduce pain [9, 20]. In a comparable intervention, Saeterbakken et al. [8] demonstrated a 49% reduction in general pain (15 mm on the VAS) without an improvement in strength after 10 weeks of training twice per week (approximately 60 min per week). The authors explained the findings in relation to different contraction forms between isometric testing and dynamic training which several others have demonstrated as well [36–38]. This may also explain the findings in the present study. Reduced muscle tension [21, 22] and pain perception [25] have also been used to explain the effects of resistance training and pain relief. The authors of the present study cannot omit that similar effects may explain the findings.

The intervention was conducted in the recruited participants’ workplaces, with an experienced instructor attending and supervising the first five training sessions. After the first week, the participants conducted the sessions independently as a social break among colleagues with a self-reported adherence of 89 and 87% for the TG10 and TG20 respectively. In a comparable study, Gram et al. [23] examined the effects of a 20-week resistance training program with supervision or minimal supervision among 351 office workers with neck- and shoulder pain. After three sessions per week, no difference between groups were observed in pain and headache [23]. In other words, using simple high-intensity exercises targeting the painful area may be more important than performing the exercises “perfect” with a close follow-up. Whether the participants were especially motivated to conduct the training program in the present study, felt an immediately positive effect or experienced a good working environment, is beyond the aim of this study. However, the participants reported a noticeable adherence in addition to improved self-reported quality of life after the training period. The improvement could be related to the pain relief, but improved work environment with social interactions during the training sessions may also interact with the reported quality of life. For example, Jakobsen et al. [39] conducted a strength training program (10 min per working day for 10 weeks) conducted at work or at home. The authors concluded workplace-based training was more effective than home-based training in reducing pain, improving strength and reducing the use of analgesics among healthcare workers.

Some limitations of the study needs to be addressed. Firstly, participants could not be blinded due to the design including two training groups. The authors cannot exclude possible non-specific effects such as the Hawthorne effect in respect to changes in pain relief. Secondly, no control group was included due to a small sample size. To compensate for this, all participants acted as their own control in the period between pre- and mid-test. This design improved the statistical power to limit the risk of producing a type II error. Importantly, the improvements in the training period had to be greater than the control period to report significant differences. Furthermore, five participants dropped out during the training period. All of the dropouts were from the group training twice per day (TG 20). One of the dropouts reported lack of time as the main reason, one was due to illness not related to the study and three did not report any reason. It is therefore possible that having two sessions within a workday is excessive and may have caused the high drop-out rate in the TG20 group. The study recruited both men (*n* = 7) and women (*n* = 23). Hormonal differences and pain experience may be influenced by sex and could influence the results. Still, the men and women were close to evenly distributed (see Table 1). Finally, we did not measure the time spent sitting during an average working day.

## Conclusion

Daily bouts of specific high-intensity resistance training of the shoulder and neck region at the workplace reduced neck- and shoulder pain and improved quality of life of office workers. However, 10 min bouts were equally effective as 2 × 10 min bouts per day. The authors recommend office workers to have one high-intensity resistance training session per day to possibly prevent and/or decrease pain in the neck- and shoulder area.

## Abbreviations

ANOVA: analysis of variance
BMI: Body mass index
EQ-5D-5 L: questioner, health-related quality of life
ES: Effect size
N: Newton
RM: repetition maximum
TG10: training group training 10 min per day
TG20: training group training 2 × 10 min per day
VAS: virtual analog scale
